# The relationship between sense of a life worth living and local self‐governance: A cross‐sectional study

**DOI:** 10.1002/jgf2.272

**Published:** 2019-08-16

**Authors:** Ryuichi Ohta, Mikiya Sato, Tetsuhiro Maeno

**Affiliations:** ^1^ Community Care Unnan City Hospital Unnan City Japan; ^2^ Department of Primary Care and Medical Education Graduate School of Comprehensive Human Sciences University of Tsukuba Tsukuba Japan; ^3^ Health Services Development and Research Center University of Tsukuba Tsukuba Japan; ^4^ Health Services Center, Occupational safety and Health Department Human Resources Group Sumitomo Heavy Industries, Ltd. Tokyo Japan; ^5^ Faculty of Medicine University of Tsukuba Tsukuba Japan

**Keywords:** citizen participation, community capacity building, life worth living, local self‐governance, rural community

## Abstract

**Background:**

Aging populations are facing increasing health problems, with social conditions often exacerbating such maladies. Although local self‐governance might effectively solve these problems, such civic participation can increase citizens’ mental and physical fatigue, which, in turn, may decrease their sense of leading productive lives.

**Methods:**

This cross‐sectional study examined the relationship between citizens’ participation in and perceptions of local self‐governance and their sense of life worth living. The study was conducted in Unnan City, which is located in the southeastern part of Shimane Prefecture. A questionnaire measuring local self‐governance and participant backgrounds was distributed. Completed questionnaires were collected between March 1 and 31, 2018. A binomial logistic regression model was used.

**Results:**

Of the distributed questionnaires, 38.5% (769/2000) were completed and returned. Responses from women and those over 65 years of age comprised 54.2% (417/769) and 35.1% (270/769) of the completed questionnaires, respectively. Analysis of the data using binomial logistic regression showed that age (≥65), health management, social interaction, learning habits, and interest in local self‐governance were significantly correlated with a sense of life worth living (odds ratio for the variables were 0.56 (*P *=* *.002), 2.58 (*P *<* *.001), 3.05 (*P *<* *.001), 2.51 (*P *<* *.001), and 1.61 (*P *=* *.009), respectively).

**Conclusion:**

Promoting local self‐governance may lead to a better sense of life worth living for citizens of rural communities. Therefore, when assessing the effectiveness of local self‐governance regarding citizens’ sense of life worth living, their interest in local self‐governance should be taken into consideration.

## Abstract 2

There is a correlation between rural citizens’ interest in local self‐governance and their sense of life worth living. Promoting local self‐governance may lead to a better sense of life worth living for citizens of rural communities.

## Graphical abstract

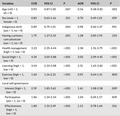

## 1 INTRODUCTION

Aging populations face social problems, such as increased demand for local health care, social isolation, and terminal care. These problems cannot always be solved by national governments,^1^ as they are often interwoven with local communities’ material conditions, culture, and history.^2^ Moreover, every community may not have the resources required to solve these problems. Therefore, some communities may not be able to implement governmental policies to solve social problems owing to a lack of both local will and resources.^3^ Local self‐governance may offer a more effective alternative to addressing social issues.

Local self‐governance refers to the self‐directed efforts of citizens to determine the conditions of their community. It may improve community well‐being by establishing solutions tailored to each community's specific problems and circumstances.^4^ The concept of local self‐governance encompasses local autonomy, community engagement, and community empowerment.^4^, ^5^ In local self‐governance, citizens act autonomously and engage in community activities, which may lead to community empowerment. Community activities include establishing local food markets to sustain the nutritional well‐being of citizens and motivating citizens to develop health‐conscious behaviors through educational forums.^6^ Citizens may then be better equipped to solve local problems and be more effective when availing themselves of local resources as compared to governmental authorities.^7^ Participating in local self‐governance and collaborating among themselves can also foster social capital and, in turn, positively affect their health and longevity.^5^, ^7^, ^8^, ^9^ This process can subsequently improve their sense of life worth living.

The sense of life worth living is the most commonly used indicator of subjective well‐being in Japan. It not only reflects an individual's well‐being but also reflects an individual's consciousness of their own motivation for living.^10^, ^11^ The sense of life worth living is essential to maintaining a healthy life and is an indicator of mental and physical well‐being.^10^ Additionally, there is a positive relationship between a sense of a life worth living and the prevention of cancer and cardiovascular diseases.^11^

Although local self‐governance can improve the health conditions of communities, it can also have negative effects, such as stress. Community members might feel pressured to improve their community and personal health; such pressure can create mental stress in citizens, which can lead to psychological disorders.^12^ Their perceptions regarding their lives, specifically sense of life worth living, may deteriorate.^13^ However, to date, no research has been conducted on the relationship between a sense of life worth living and citizens’ involvement in local self‐governance. Clarification of the relationship can lead to further consideration and the effective provision of local self‐governance to improve citizens’ health conditions. Therefore, the present study investigated whether a relationship exists between interest, action, and realization in local self‐governance and a community member's sense of life worth living.

## 2 MATERIALS AND METHODS

To explore this relationship, we performed a cross‐sectional study using data collected via a questionnaire on citizens’ attitudes toward local self‐governance and their health and lifestyle. The study was conducted in Unnan City, which is located in the southeastern part of Shimane Prefecture in Japan. This city has 30 autonomous communities. Each community engages in various activities pertaining to local self‐governance. These activities include self‐managed agricultural and industrial stores that sell their own products, regular educational meetings on medical conditions, exercise programs for elderly citizens, and programs monitoring the conditions of isolated elderly people. Provisions of these or new activities regarding local self‐governance are discussed and decided in each community's regular meetings.

Unnan City has a population of approximately 40 000. Adults over 65 years of age comprise over 33% of the total population, and this number is expected to exceed 50% of the total population by the year 2025. Participants in the present study included citizens of Unnan City who were over 20 years of age and were randomly selected from the city's registry of citizens. Further, the demographic characteristics of 2000 citizens were extracted from the city's Resident Registration System (jumin‐kihon‐daicho).

### 2.1 Data collection

The self‐administered questionnaire asked participants about their demographic characteristics, place of birth,^6^, ^13^ perceived level of local self‐governance, sense of life worth living,^11^ and factors related to their health. In March 2018, this questionnaire was mailed to and collected from participants. To increase the response rate, the front page of the questionnaire explained the importance of this research for clarifying present community activities and constructing future ones.

### 2.2 Dependent and independent variables

In the absence of well‐developed scales to measure different aspects of local self‐governance, we created an original scale with three items. Each item had a four‐point Likert response scale, ranging from “not at all” to “strongly agree” in order to measure the participants’ interest in, actual participation in, and perception of the effectiveness of local self‐governance. Based on previous studies on local autonomy and community engagement,^4^, ^5^ we created Questions 1, 2, and 3 to assess the participants’ interest in, actual participation in, and perception of the effectiveness of local self‐governance, respectively:

1. Are you interested in the activities of your local community?
2. Have you participated in community activities at least once this year?
3. Do you think that the community is solving its problems autonomously?

To measure citizens’ perceptions of and behaviors related to their health and personal lives, we asked them various questions, based on the previous research, about their perception of sense of life worth living.^11^ We also asked them about their educational and exercise habits,^11^ whether they had a primary care physician,^14^ their health management,^15^ and their relationships with neighbors (Table 1).^16^ Responses were obtained on a four‐point Likert scale from “not at all” to “strongly agree,” except for the questions pertaining to having a primary care physician, sex, age, place of birth, and participation in local self‐governance.

**Table 1:** Contents of the questionnaire

Code	Contents
Age	Are you over 65 y old?
Sex	Please tell me your sex.
Indigenous birth	Were you born in this town?
Sense of a life worth living	Do you feel you have a life worth living?
Primary care doctor	Do you have a primary care doctor?
Health management	Do you actively manage your health?
Social	Does your community provide social support for maintaining citizens’ health?
Learning	Do you learn something new each day?
Exercise	Do you exercise regularly?
Local self‐governance	
Interest	Are you interested in participating in community activities?
Participation	Have you participated in community activities more than once in the previous year?
Effectiveness	Do you feel that your community can solve its problems by itself?

### 2.3 Statistical analysis

We categorized all ordinal variables as binomials (high to mild = 1, low to not at all = 0). For categorical data, we used the chi‐square test. A binary multiple regression model was employed to assess the sense of life worth living, which was adjusted for age; sex; birthplace; the participants’ interest in, actual participation in, and perception of the effectiveness of local self‐governance; having a primary care physician; health management; social interaction; learning habits; and exercise habits. We analyzed the data using Stata 14 (Stata Corp.).

### 2.4 Ethical considerations

Participants were informed that the data collected by the questionnaire would be used for research purposes alone. Further, questionnaire data were anonymized. In the instructions to the questionnaire, we explained the aims of the research, the type of data to be disclosed, and how their personal information would be protected. In addition, the instructions clearly stated that by answering the questionnaire, participants were effectively consenting to participate in the study. This study was approved by the Unnan City Hospital Clinical Ethics Committee (the approval number 20180013).

## 3 RESULTS

Among the distributed questionnaires, 38.5% (769/2000) were completed and returned, as the questionnaires lacking data were omitted from the analysis. Responses from women and those older than 65 years of age comprised 54.2% (417/769) and 35.1% (270/769) of the completed questionnaires, respectively, and 73.9% (569/769) reported that they were born in Unnan City. Those with primary care physicians accounted for 76.2% of the participants, and 68% reported that they usually felt a sense of life worth living. More than 60% of the participants managed their health by themselves and had daily social interactions with neighbors. Approximately 30% of the participants exercised and practiced some form of self‐education on health topics. More than 70% of the participants were interested in and participated in local self‐governance; however, <40% of all the participants recognized its effectiveness (Table 2).

**Table 2:** Demographic data of the study's participants

Variables	N = 769/2000
Age ≥ 65, N (%)	270 (35.1)
Female sex, N (%)	417 (54.2)
Indigenous birth, N (%)	569 (73.9)
Having primary care physician, N (%)	586 (76.2)
Sense of a life worth living, strongly to moderately agree, N (%)	523 (68.0)
Health management, strongly to moderately agree, N (%)	499 (64.9)
Social, strongly to moderately agree, N (%)	532 (69.2)
Learning, strongly to moderately agree, N (%)	240 (31.2)
Exercise, strongly to moderately agree, N (%)	291 (37.8)
Local self‐governance interest, strongly to moderately agree, N (%)	567 (73.7)
Local self‐governance participation, Yes, N (%)	542 (70.5)
Local self‐governance effectiveness, strongly to moderately agree, N (%)	306 (39.8)

Abbreviation: N, sample size.

Results of the binomial logistic regression model showed that age (≥65), health management, social interaction, learning habits, and interest in local self‐governance were significantly correlated with a sense of life worth living [odds ratio for the variables were 0.56 (*P *=* *.002), 2.58 (*P *<* *.001), 3.05 (*P *<* *.001), 2.51 (*P *<* *.001), and 1.61 (*P *=* *.009), respectively] (Table 3).

**Table 3:** Association between participants’ sense of a life worth living and demographic variables

Variables	COR	95% CI	*P*	AOR	95% CI	*P*
Age (≥65 = 1, 65 ≥ 0)	0.93	0.87‐1.00	.067	0.56	0.38‐0.81	.002
Sex (male = 1, female = 0)	0.85	0.63‐1.16	.321	0.70	0.49‐1.01	.059
Indigenous birth (yes = 1, no = 0)	0.89	0.79‐1.01	.064	0.98	0.66‐1.47	.941
Having a primary care physician (yes = 1, no = 0)	1.79	1.27‐2.53	.001	1.28	0.85‐1.93	.233
Health management (high = 1, low = 0)	3.23	2.35‐4.44	<.001	2.58	1.76‐3.79	<.001
Social (high = 1, low = 0)	4.24	3.05‐5.88	<.001	3.05	2.09‐4.45	<.001
Learning (high = 1, low = 0)	3.44	2.33‐5.08	<.001	2.51	1.65‐3.82	<.001
Exercise (high = 1, low = 0)	1.60	1.16‐2.21	<.001	0.95	0.64‐1.41	.800
Local self‐governance						
Interest (high = 1, low = 0)	2.59	1.85‐3.61	<.001	1.61	1.08‐2.38	.009
Participation (yes = 1, no = 0)	1.86	1.34‐2.54	<.001	1.04	0.69‐1.57	.839
Effectiveness (high = 1, low = 0)	1.80	1.31‐2.49	<.001	1.13	0.78‐1.64	.516

Abbreviations: AOR, adjusted OR; CI, confidence interval; COR, crude OR; OR, odds ratio; P, *P*‐value.

## 4 DISCUSSION

The results of the present study revealed that citizens’ interest in local self‐governance was positively correlated with a sense of life worth living. However, no statistical relationships were found between citizens’ sense of life worth living and their participation in and perception of the effectiveness of local self‐governance. In addition, attitudes toward health among the members of a community were also related to a sense of life worth living.

According to our results, interest in local self‐governance was related to citizens’ sense of life worth living, regardless of their participation in and perception of the effectiveness of local self‐governance. The idea of local self‐governance is often introduced into various local communities, particularly in rural areas.^17^, ^18^ Procedures encouraging citizens to become interested in local self‐governance can help improve citizens’ sense of life worth living. However, to solve their problems effectively, citizens must be highly knowledgeable about their communities.^14^, ^17^, ^18^ Local self‐governance activities may encourage citizens to learn new things about their communities, which can lead to increased feelings of burden and consequent stress.^19^, ^20^ This negative potential trajectory may be related to the lack of relationship found in this study between a sense of life worth living and citizens’ participation in and perception of the effectiveness of local self‐governance. When engaging in local self‐governance, citizens must be sure to address the accompanying pressure and stress.

Citizens’ health behaviors may be related to whether or not they feel a sense of life worth living. In this study, citizens who positively managed their health reported high levels of having a sense of life worth living. This result matches the results of previous studies that found a correlation between a sense of life worth living and healthy behaviors and younger generations.^11^, ^21^ In addition, social interaction may positively affect citizens’ sense of life worth living.^15^ Citizens with high social capital interact widely with others in their community, which increases their access to useful health information.^22^

Access to a learning environment may also contribute to social capital.^23^ Citizens with regular educational opportunities and daily access to places where they may learn something new feel increased satisfaction. This increased satisfaction, in turn, increases their sense of life worth living.^24^ Various factors contribute to the development of one's sense of life worth living, all of which should be considered when promoting local self‐governance within a community. Moreover, active participation is driven by the participants’ interests, which should not be imposed on them by forces external to the community.^25^ Therefore, particularly in rural communities, focusing on citizens’ interests in local self‐governance will be conducive to the promotion of such activities.

This study is the first to demonstrate a correlation between rural citizens’ interest in local self‐governance and their sense of life worth living. Rural citizens who are interested in local self‐governance may live with a high sense of life worth living, regardless of their participation and their perception of the effectiveness of local self‐governance. Therefore, when assessing the effectiveness of local self‐governance regarding citizens’ sense of life worth living, their interest in local self‐governance should be taken into consideration.

### 4.1 Limitations and recommendations

One limitation of this study is the cross‐sectional design, which prevented the establishment of a causal relationship between a citizen's sense of life worth living and other independent variables. However, this study revealed a relationship among the aforementioned factors. Therefore, future studies should examine the effects of an increase in the interests, participation, and perception of the effectiveness of local self‐governance on health outcomes. Another confounding factor might exist, which may affect the internal validity. As potential confounders, age, sex, and place of birth were measured, but socioeconomic status was not. Further research should focus on other confounding factors related to citizens’ mutable characteristics, such as body weight, level of education, and financial conditions.

A further limitation is the low response rate to the questionnaire, which can affect the external validity of the study. Although this questionnaire was provided randomly to citizens, empowered citizens are more likely to have answered the questionnaire because of their interest in local self‐governance and active participation in their communities. This might reduce this study's external validity.

In addition, our questionnaire on local self‐governance may require further validation. Since no extant qualified questionnaires were available on the study topic, we designed a new questionnaire to assess the interest, participation, and perception of effectiveness of local self‐governance. Future research should validate this questionnaire by using it in various studies.

## CONFLICTS OF INTEREST

The authors have stated explicitly that there are no conflicts of interest in connection with this article.

## ETHICAL APPROVAL

This study was approved by the Unnan City Hospital Clinical Ethics Committee.
